# pH-Responsive Inorganic/Organic Nanohybrids System for Controlled Nicotinic Acid Drug Release

**DOI:** 10.3390/molecules27196439

**Published:** 2022-09-29

**Authors:** Seungjin Yu, Huiyan Piao, N. Sanoj Rejinold, Hanna Lee, Goeun Choi, Jin-Ho Choy

**Affiliations:** 1 Department of Nanobiomedical Science and BK21 PLUS NBM Global Research Center for Regenerative Medicine, Dankook University, Cheonan 31116, Korea; 2 Intelligent Nanohybrid Materials Laboratory (INML), Institute of Tissue Regeneration Engineering (ITREN), Dankook University, Cheonan 31116, Korea; 3 College of Science and Technology, Dankook University, Cheonan 31116, Korea; 4 Division of Natural Sciences, The National Academy of Sciences, Seoul 06579, Korea; 5 Department of Pre-Medical Course, College of Medicine, Dankook University, Cheonan 31116, Korea; 6 International Research Frontier Initiative (IRFI), Institute of Innovative Research, Tokyo Institute of Technology, Yokohama 226-8503, Japan

**Keywords:** nicotinic acid, reduced side effects, layered double hydroxide, Eudragit^®^ S100, drug release, cyto-compatibility

## Abstract

Although nicotinic acid (NA) has several clinical benefits, its potency cannot be fully utilized due to several undesirable side effects, including cutaneous flushing, GIT-associated symptoms, etc. To overcome such issues and improve the NA efficacy, a new inorganic–organic nanohybrids system was rationally designed. For making such a hybrid system, NA was intercalated into LDH through a coprecipitation technique and then coated with Eudragit^®^ S100 to make the final drug delivery system called Eudragit^®^ S100-coated NA-LDH. The as-made drug delivery system not only improved the NA release profile but also exhibited good bio-compatibility as tested on L929 cells. Such an inorganic–organic nanohybrid drug delivery agent is expected to reduce the undesirable side effects associated with NA and hopefully improve the pharmacological effects without inducing any undesirable toxicity.

## 1. Introduction

Nicotinic acid (NA), chemically C₆H₅NO₂, otherwise called niacin or vitamin B3, is a hydrophilic vitamin and the oldest extensively used hypolipidemic drug since 1955 [1]. It is structurally close to nicotinamide, a precursor of coenzymes NAD (nicotinamide adenine dinucleotide)/NADP (nicotinamide adenine dinucleotide phosphate), which is a main electron acceptor in the oxidation reaction of fuel metabolites [2]. Based on the literature, only nicotinic acid, not nicotinamide, has pharmaceutical benefits and has been utilized for treatment of dyslipidemic states [1]. Upon consuming a low concentration (typically milligrams) of NA, it could act as a vitamin in our body, whereas a higher dose (typically grams) would result in clinical benefits for treating dyslipidemia, which is effective for reducing low-density lipoprotein (LDL) cholesterol levels and simultaneously enhancing healthy cholesterol and high-density lipoprotein (HDL) cholesterol levels. Moreover, NA has an attenuation effect of oxidative stress [3] of chronic renal failure, as demonstrated in a rat model [4]. It was experimentally evidenced that supplementation with NA may provide mitigation of oxidative stress and hepatoprotective potential against an overdose of acetaminophen [5].

Although NA has many clinical benefits [6,7,8], the major concerns are associated with its not severe but undesirable side effects, primarily a cutaneous symptom, such as flushing, which is from strong vasodilation and occasionally causes a burning sensation, GI symptoms and the necessity for multiple daily administrations of the drug [9]. Although flushing is not that harmful, it prevents long-term administration of the drug for many patients [10]. Previous reports suggest that the flushing happens at a lower dose of 50 mg/d, but, in reality, the therapeutic dosage window for NA is much higher than this [9]. According to a previous experiment, almost 100% of the participants experienced vasodilatory side effects when taken in an immediate-release (IR) dosage, and, eventually, 25% of the patients could not continue NA use because of these disadvantages [11]. 

Additionally, it was reported that oral administration of NA in human gut rapidly absorbed with fast blood clearance in 1.5–2 h. According to the experiment, 1 g of orally administered NA had an improved plasma level of free NA (26–28 µg/mL), which was rapidly cleared after 6 h. Moreover, the plasma level of NA showed no elevation, and the pharmacological effects and even the drug disappeared in less than an hour after infusion being inactivated [12,13]. Additionally, NA-IR required a multiple-dosage regimen to meet the required efficacy (typically two or three times a day) [14,15,16].

Generally, NA can be available in immediate-release (IR) or sustained-release (SR). NA-IR usually takes 1 to 2 h to be fully absorbed, while the SR one has different absorption rates, which can vary up to 12 h or more than that [17]. Based on the conventional formulations, only use of immediate-release (IR) niacin shows NA or NA-flushing and limitations [13,17]. These side effects depend on how immediately NA could be absorbed and metabolized after being delivered from the different products [17].

Further, the side effects of NA could be worse if it has shorter T_1/2_ [18]. Our mucoadhesive formulation of niacin is anticipated to have reduced side effects owing to its controlled release capability. 

The main goal of SR formulations of NA is to have reduced flushing due to its preferential metabolism by the nicotinamide pathway [19,20,21]. To achieve this, slow-release formulations of NA are suggested. Generally, SR formulations slow down the intestinal release of NA and reduce the C_max_ compared to intact NA, reducing the flushing phenomenon and lowering the GI side effects [15]. It was observed that patients dosed with IR niacin exhibited more flushing (~53% of patients) than those treated with SR niacin (22%) [21]. 

Importantly, oral administration of NA can have side effects, such as flushing, and gastrointestinal troubles, such as nausea and diarrhea [22]. Flushing can last for 0.5–1.5 h, accompanied by intense erythema, along with skin irritation and hyperthermic skin condition as well [23]. In certain patients, these symptoms can even be worse, with burning sensation, urticaria, periorbital edema, conjunctivitis or nasal congestion. More than 30% of patients treated with NA stopped the therapy due to the aforementioned side effects [24]. Such adverse reactions are also associated with how they are delivered to various organs from the carrier systems [17,25].

The problems mentioned above are all related to the NA’s bioavailability [9]. To enhance and overcome these situations, an inorganic–organic hybrid drug delivery system is proposed in this study. The inorganic material is based on layered double hydroxide (LDH) due to its efficiency in controlling drug release at the molecular level in a sustained manner. LDH, anionic clay, has a positively charged hydroxide lattice, with the brucite structure and replaceable hydrated anions and H_2_O molecules in the interlayer [26,27,28,29,30,31,32,33,34]. The LDH drug delivery system has been attracting the scientific community owing to its ease of preparation, improved drug bioavailability, stability, better pharmacological performance with no toxicity and, most importantly, good controllability on drug release in a sustained fashion [35,36,37,38,39,40,41,42,43,44,45]. The general formula of LDHs is expressed as [M^2+^_1–x_- M^3+^_x_(OH)_2_]^x+[^A_n_^−^_x_/n]·mH_2_O, where M^2+^ is a divalent cation, such as Ca^2+^, Zn^2+^, Co^2+^, Ni^2+^, Mn^2+^, etc., M^3+^ is a trivalent cation, such as Al^3+^, Fe^3+^, Cr^3+^, V^3+^, etc., and A^n−^ is an interlayer anion, such as inorganic NO_3_^–^, CO_3_^2–^, SO_4_^2–^ or Cl^−^, or organic ion, such as ursodeoxycholic acid [46], nucleosides, DNA, etc. [47,48].

The organic part was based on Eudragit^®^ S100, one of the methacrylic acid copolymers, consisting of methacrylic acid: methyl methacrylate, which has been utilized very well for drug delivery applications [49,50,51,52,53,54,55,56,57,58,59,60,61,62,63,64,65,66,67]. Moreover, the FDA has approved and included various kinds of methacrylic acid copolymers, including Eudragit^®^ S100, in the list of “Inactive Ingredient Search for Approved Drug Products” [68]. It has a pH-responsive dissolution property under pH 5–7 due to the carboxylic acid that can be changed to the carboxylate group [69]. Therefore, it could be used as enteric coatings for controlling burst release under acidic conditions because Eudragit^®^ S100 can only be swollen under a neutral pH [70]. By making an inorganic–organic hybrid system, Eudragit^®^ S100 can prevent sudden decomposition under acidic conditions of GIT.

**Scheme 1:**
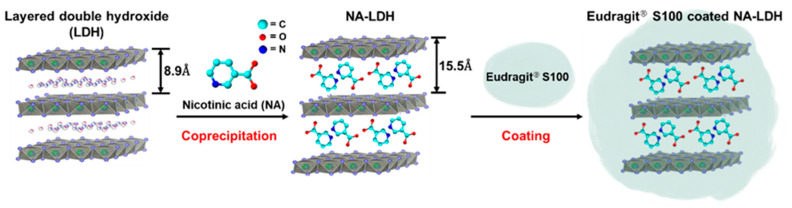
Schematic for the NA molecules intercalated in the LDH layer via coprecipitation reaction.

Here, an NA-based pH-responsive drug delivery system [71] was rationally engineered, in which NA molecules are intercalated in the interlayer space of LDH, which were then coated with Eudragit^®^ S100 for restricting the fast decomposition of LDH under an acidic environment (Scheme 1).

Therefore, the present manuscript will detail the synthesis and characterization of thus made inorganic–organic nanohybrids of nicotinic acid by powder XRD, ICP, Fourier transform infrared analysis, FE-SEM, DLS, Zeta, UV–Vis spectrophotometer and TGA to study the characteristic of NA immobilized in the LDH. In addition, the release profiles of NA with intact NA, non-coated and coated nanohybrids will also be detailed under simulated gastric/intestinal fluids. Further, in vitro cyto-compatibility using L929 cells confirmed the biocompatibility of the developed hybrids even at high concentration, confirming their suitability as an effective NA delivery platform for sustaining the nicotinic acid drug release. 

## 2. Results

### 2.1. Powder X-ray Diffraction Analysis

**Figure 1:**
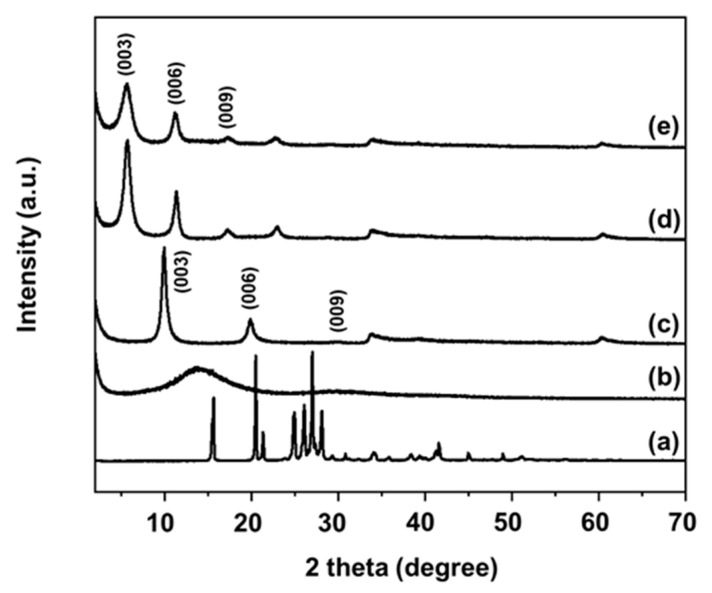
PXRD patterns of (a) intact NA, (b) Eudragit^®^ S100, (c) pristine LDH, (d) NA-LDH and (e) Eudragit^®^ S100-coated NA-LDH.

The powder X-ray diffractometer patterns of (a) intact NA, (b) Eudragit^®^ S100, (c) pristine LDH, (d) NA-LDH nanohybrid and (e) Eudragit^®^ S100-coated NA-LDH are shown in Figure 1, demonstrating well developed (*00l*) reflections, such as (*003*), (*006*), (*009*). Since LDH has a lamellar structure, crystallographic positions (*00l*) could provide information regarding the d-value [72]. The pristine LDH showed a characteristic sharp (*003*) peak at around 9.95°, representing the interlayer distance of 8.90 Å, indicating the immobilization of an NO_3_^−^ anion. As per Bragg’s law, however, the basal spacing of NA-LDH and Eudragit^®^ S100-coated NA-LDH, 15.5 Å, considerably shifted after successful intercalation of NA molecules to be a single-layer arrangement into the metal layers. There were no peaks of crystalline NA after hybridization, suggesting that NA was totally dissolved and intercalated into the interlayer space at the molecular level. The ICP data in Table S1 also described that the Zn/Al molar ratios of pristine LDH and NA-LDH are 2.08 and 2.01, respectively. Moreover, no different XRD patterns between non-coated NA-LDH and coated NA-LDH indicate that NA still remained in the LDH space even after Eudragit^®^ S100 coating. Regarding the layer thickness of the Zn_2_Al_1_-LDH layer (4.8 Å) and the d-value of NA-LDH (15.5 Å), the gallery height of NA-LDH could be calculated to be 10.7 Å. Consequently, the intercalative NA molecules have a tilted bilayer arrangement to create a stable arrangement, calculating the tilting angle of about 73.7° (Scheme 1). 

### 2.2. FT-IR Studies

**Figure 2:**
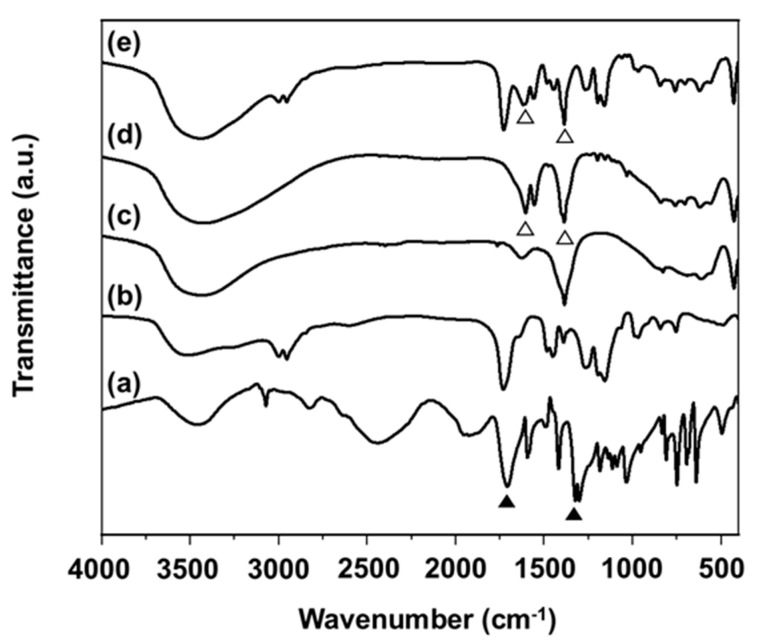
FT-IR spectra of (a) intact NA, (b) Eudragit^®^ S100, (c) pristine LDH, (f) NA-LDH and (e) Eudragit^®^ S100-coated NA-LDH.

Various samples, such as NA, Eudragit^®^ S100, pristine LDH, NA-LDH and Eudragit^®^ S100-coated NA-LDH, were measured for FT-IR (Figure 2). The characteristic bands of intact NA are broad bands at 3450 [73] and sharp ones at 3073 and 2830 cm^−1^ due to the O-H and C-H stretch modes. FT-IR shows two major peaks associated with carboxylic acid at 1710 and 1320 cm^−1^ due to asymmetric and symmetric bonds (▲, black up-pointing triangle) [74,75,76]. As the NaOH is titrated up, NA could be deprotonated and intercalated in the LDH interlayer, so the peaks related to -COOH would be changed. 

For pristine LDH, a broad band region at 3400 cm^−1^ and 1626 cm^−1^ can be ascribed to O-H stretching vibrations owing to the -OH functionalities of the LDH and the water contents in the interlayers of LDH. The intense and sharp band at 1384 cm^−1^ is due to the nitrate vibration in the interlayers. The low frequency peaks appearing in the range of 830–425 cm^−1^ are attributed to the M-O and M-O-M vibrations of the LDH lattice [77]. For Eudragit^®^ S100, the broad peaks around 3300–3600 cm^−1^ and 2900–3000 cm^−1^ are due to (O-H), (C-H) groups, respectively [77]. In all products, including LDH, the broad band region 2500–3500 cm^−1^ located in every spectrum is due to O-H stretching absorption, and the characteristic stretching vibration of LDH at 830 and 425 cm^−1^ appeared. As mentioned above, changed peaks of NA-LDH and Eudragit^®^ S100-coated NA-LDH are found at 1608 and 1400 cm^−1^, which are due to the asymmetric vibration of ν_as_(COO^−^) and the symmetric vibration of ν_s_(COO^−^), respectively (∆, white up-pointing triangle) [78,79]. All the characterized bands from NA have been overlapped in the spectra of NA-LDH and Eudragit^®^ S100-coated NA-LDH, confirming that the drug could be well stabilized within the LDH carrier via electrostatic bonding, with no major changes to the intrinsic NA. We have shown characteristic peaks mentioned above in Table S2.

### 2.3. FE-SEM and DLS Analysis 

Based on the FE-SEM studies, Figure S1 shows the morphology of Eudragit^®^ S100-coated NA-LDH. The Eudragit ^®^ S100 spray-coated samples (Eudragit^®^ S100-coated NA-LDH) observed to have a spherical shape were of ~5 µm average size (Figure S1), which was much larger than that of the non-coated NA-LDH particle. According to the DLS results, the average particle sizes of pristine LDH, NA-LDH and Eudragit^®^ S100-coated NA-LDH were found to be 273.4 ± 71.9, 212.5 ± 59.4 and 4569.6 ± 457.5 nm (Figure S2), respectively. The micron size of Eudragit^®^ S100-coated NA-LDH from the DLS result was in good agreement with the FE-SEM image. This result describes that the plate-like NA-LDH particles were well coated with Eudragit^®^ S100 to form a larger spherical morphology through the spray coating method [80].

### 2.4. Surface Charge

Surface charges of pristine LDH, NA-LDH and Eudragit^®^ S100-coated NA-LDH were analyzed using zeta potentials in an aqueous solution with pH 7 (Table S3). Based on the zeta-potential analysis, pristine LDH was cationic in charge (+40.9 mV), with narrow distribution. Compared with pristine LDH, zeta potential values of NA-LDH showed almost neutral charges centered at −0.6 mV. The hybrid particles had different zeta potential values to the neutral region, meaning that charge neutralization occurred in the sample after intercalation [81]. After Eudragit^®^ S100 coating, Eudragit^®^ S100-coated NA-LDH has a negative charge of −21.5 mV, which is correlated to the negative charge in the neutral condition attributed to free acrylic acid groups of anionic polymer [82,83].

### 2.5. Determination of NA Content

The content of NA was analyzed with non-coated NA-LDH and Eudragit^®^ S100-coated NA-LDH using a UV–Vis spectrophotometer. The contents of NA in NA-LDH and coated NA-LDH were 26.1 ± 0.7% and 10.9 ± 0.7%, respectively, which were appropriate in relation to the theoretical contents of 30.5% and 12.2%, respectively. The Eudragit^®^ S100 coating (mass fraction of nanomaterials and Eudragit, 1:1.5, *w*/*w*) enabled the nanohybrids to have a decreased NA content compared to that of the uncoated ones.

### 2.6. In Vitro Release Study

We expected that the NA would release at a slow rate after oral medication compared to other candidates. We tried to employ an intercalation method for delaying the release rate through inorganic materials, LDH. LDH has been often utilized as a nanocarrier due to its ability to release drugs in a sustained manner. Here, we expect that LDH could enable improved intestinal absorption, which can be further boosted with additional coating of Eudragit^®^ S100. 

**Figure 3:**
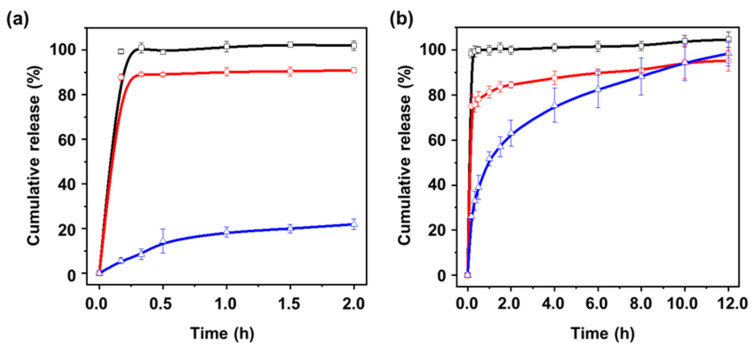
In vitro release profiles of NA from intact NA (─□─), NA-LDH (─○─), Eudragit^®^ S100-coated NA-LDH (─△─) (**a**) pH 1.2 medium and (**b**) pH 6.8 one.

We investigated the sustained release properties (Figure 3) of NA molecules of intact NA, NA-LDH and Eudragit^®^ S100-coated NA-LDH under simulated gastric solution (pH 1.2). According to the NA release behavior, as expected, almost all the NA was rapidly dissolved from intact NA and NA-LDH in the initial 30 min (≈100%). In the case of LDH, the protons attacked and destroyed the layered structure in acidic medium, leading to release of the intercalation molecules in its amorphous ionic type. With Eudragit^®^ S100-coated NA-LDH, however, Eudragit^®^ S100 functioned as a diffusion barrier by blocking the dissolution of LDH at a low pH. Therefore, regarding the release rate of Eudragit^®^ S100-coated NA-LDH after 2 h, it reached about 20%, which is much lower than NA and NA-LDH. 

We also tested the release profiles of NA in the intestinal condition of pH 6.8. As the pH increased, the dissolution of NA of Eudragit^®^ S100-coated NA-LDH became remarkable in terms of the controlled release pattern compared to other samples. At pH 6.8, the release tendency of intact NA is almost the same as pH 1.2, which is released in an instant within 30 min (≈100%). However, NA-LDH showed much slower release than in the gastric condition, which is because LDH is known not to dissolve in the basic media. Based on the literature, Eudragit^®^ S100, an anionic polymer, at neutral pH, not only could replace the immobilized molecule, NA, efficiently by ion exchange reaction but also utilize the expanding of the LDH’s interlayer distance. Therefore, Eudragit^®^ S100-coated NA-LDH would easily disperse in the intestinal solution. 

### 2.7. Kinetic Model

To have a complete understanding regarding the NA drug release profile from the NA-LDH and Eudragit^®^ S100-coated NA-LDH, the observed release profiles were fitted to four kinetic models as plotted in Figure S3, and the obtained rate constant values (k_d_) and r^2^ were calculated in Table S4. Considering the r^2^ value, the release curves of NA from NA-LDH hybrids and Eudragit^®^ S100-coated NA-LDH were from the best fitted given in Table S4. Out of the various kinetic models, the release profiles were best fitted to parabolic and Elovich diffusion models, respectively. The calculated r^2^ values of NA-LDH are 0.9238 and 0.9774, and the case of Eudragit^®^ S100-coated NA-LDH are 0.9106 and 0.9930. According to the parabolic diffusion model, the guest molecules from the lattice could be released either due to interparticle diffusion or surface diffusion. The other suited model, Elovich, described various release processes, including surface diffusion [84]. Based on all the summarized results, it was concluded that NA release from the nanohybirds is regulated by a diffusion mechanism as the coating agent as well as the core, LDH, and both are highly pH-sensitive bioactive materials [84].

### 2.8. TG Analysis

The TGA and DTA results of LDH, NA-LDH and Eudragit^®^ S100-coated NA-LDH are shown in Figure S4. All the samples, including pristine LDH, NA-LDH and Eudragit^®^ S100-coated NA-LDH, have two steps of weight loss as follows: the first half is ~260 ℃ and represents the desorption of water molecules on the outer surface and interlayer water of the pristine LDH (25.2%), NA-LDH (26.5%) and Eudragit^®^ S100-coated NA-LDH (13.9%). This information is in accordance with the DTA results shown in Figure S4d. The second half weight reduction of pristine LDH was in between 260 °C and about 500 °C, corresponding to the dehydroxylation of LDH. In the case of NA-LDH, second weight loss could be for two reasons: (1) decomposition and combustion of intercalated NA anion in LDH lattice, and (2) dehydroxylation of LDH, leading to large mass reduction of NA-LDH between 260–500 °C compared the pristine one [77]. However, in the case of Eudragit^®^ S100-coated NA-LDH, it had the highest weight loss in the second step due to additional effects of NA-LDH and the organic coating agent.

### 2.9. Cytotoxicity Studies of the LDH Nanohybrids 

**Figure 4:**
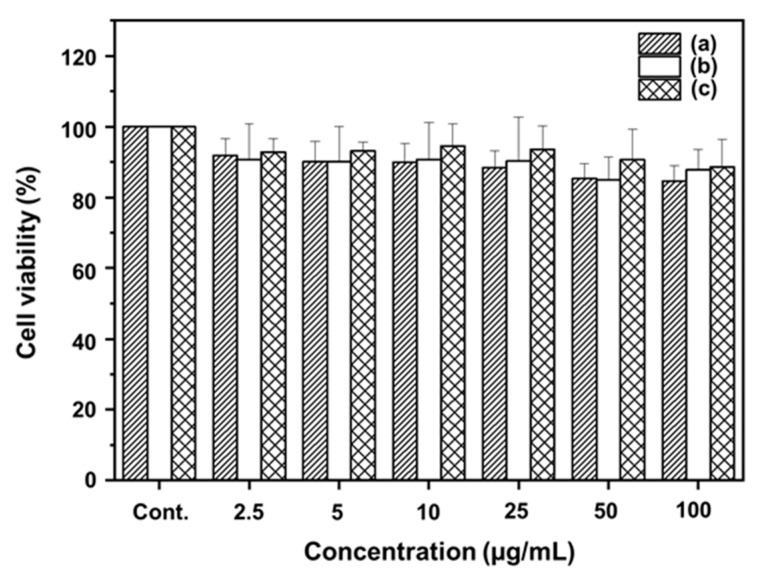
MTT assay of L929 cells following 24 h of treatment with (a) NA, (b) pristine LDH, and (c) NA-LDH.

Cytotoxicity is one of the major concerns when using LDH nanoparticles in biomedical applications, and it has been well known for its non-toxicity even at higher concentrations. Here, all the samples, such as NA, pristine LDH and NA-LDH, were non-toxic even at a higher concentration of 100 µg/mL. There were no obvious differences between the samples, as shown in Figure 4.

## 3. Discussion

A previous study by Kleyi et al. (2021) [85] reported the Zn/Al LDH nanostructure as an efficient topical delivery vehicle for NA. However, careful XRD observation suggested that the phase involved no single crystals, unlike ours. Even though they claimed pH-responsive drug release behavior, its pH dependency between acidic and neutral pH was not dramatically different, mainly due to the lack of a proper coating. However, we used an inorganic–organic nanohybrid based on LDH and Eudragit^®^ S100, where the sudden decomposition of the former can be controlled by the latter’s coating, which is important to have effective GIT stability, thereby achieving maximum therapeutic efficacy under a neutral pH environment in the intestinal sites.

In an earlier work (2019), NA was loaded into LDH with varying ions in the interlayer spaces with different Mg/Al ratios, and it was observed that NO_3_-LDH 2:1, with a 2:1 Mg/Al ratio (with NO_3_^−^ ions), has the best NA adsorption capacity [86].

Most of the previous attempts were not focused on developing a controlled release system for NA, whereas our inorganic–organic nanohybrids were able to have controlled nicotinic acid release, which is very important in reducing the flushing and gastro-intestinal side effects. Additionally, such well-engineered NA nanohybrids could be further utilized for various applications related to cosmeceutical formulations with improved efficacy in the near future.

## 4. Materials and Methods

### 4.1. Materials

Nicotinic acid (C_6_H_5_NO_2_), zinc nitrate hexahydrate (Zn(NO_3_)_2_·6H_2_O, purity > 98.0%), aluminum nitrate nonahydrate (Al(NO_3_)_3_·9H_2_O, purity > 98.0%) and sodium hydroxide (NaOH) were purchased from Aldrich (Seoul, Korea). Eudragit^®^ S100 (MW = 125,000 g/mol) was supplied by Evonik (Essen, Germany). 

### 4.2. Synthesis of the NA-LDH Nanohybrid

#### 4.2.1. NA-LDH Nanohybrid 

It was prepared by a conventional coprecipitation method. Powdered NA (0.81 g) was first treated with decarbonated water (50 mL) and titrated with NaOH solution (0.5 M) to produce Na-salt type NA solution at pH 8.0. 50 mL of mixed metal solution containing Zn(NO_3_)_2_ · 6H_2_O (1.96 g) and Al(NO_3_)_3_ · 9H_2_O (1.24 g) with molar ratio of Zn/Al = 2/1 was then added to the NA solution, and it was then titrated with NaOH solution (0.5 M) until pH ~8.0 to produce white precipitates. The prepared suspension was stirred maintaining temperature at 40 °C for 24 h. The reactions were completed in an N_2_ environment for preventing any carbonate contamination from the surroundings. The resulted precipitation was centrifuged, given 3 times washing with decarbonated water and finally freeze-dried. The above procedure was also carried out without NA solution to prepare LDH carriers only (mentioned as pristine LDH). 

#### 4.2.2. Eudragit^®^ S100-coated NA-LDH

To prepare the Eudragit^®^ S100-coated NA-LDH, the NA-LDH(0.8 g) was dispersed in EtOH (95%) (10 mg/mL), where Eudragit^®^ S100 (1.2 g) (10 mg/mL) was dissolved in the same solvent (weight ratio of Eudragit^®^ S100:NA-LDH = 1.5: 1.0). Further, it was coated by spray drying technique (SD-06A, Labplant, North Yorkshire, UK) as follows: inlet temperature of 95 °C, outlet temperature of 40–50 °C, pump flow rate of 2115 mL/h and drying air speed of 4.3 m/s).

### 4.3. Sample Characterization 

The PXRD patterns for all the samples were determined by Bruker D2 Phase diffractometer (Bruker, Karlsruhe, Germany) equipped with Cu Kαradiation (λ = 1.5418 Å). The tube voltage and the current were maintained at 30 kV and 10 mA, respectively. For FT-IR analysis, a Jasco FT/IR-6100 spectrometer (Jasco, Tokyo, Japan) machine was used and a traditional KBr pelleting technique was used. The spectral range was 4000–400 cm^−1^ and resolution of 1 cm^−1^ with 40 scans per spectrum were set for data acquisition. The morphologies for Eudragit^®^ S100-coated NA-LDH were studied by field emission scanning electron microscopy (JEOL-6700F, JEOL Ltd., Tokyo, Japan). Surface charge and particle size of pristine LDH, NA-LDH and Eudragit^®^ S100-coated NA-LDH were measured using zeta potentiometer (ELSZ-2000ZS; Otsuka, Tokyo, Japan) in distilled water, respectively. To determine the metal molar ratio of the hybrids, inductive coupled plasma (ICP) analysis (OPTIMA 8300 (Perkin-Elmer, Waltham, MA, USA)) was performed. The thermogravimetric and differential thermal analyses (TG-DTA) (TG8121, RIGAKU, Tokyo, Japan) were performed under airflow with a heating rate of 10 °C/min in the temperature range from 30 °C to 800 °C. 

### 4.4. Determination of NA Content

To determine the encased NA amount of NA-LDH hybrids and Eudragit^®^ S100-coated NA-LDH, each sample (5 mg of NA-LDH hybrids) was dispersed in 0.1 M HCl aqueous solution, 65% ethanol (95%) solution and sonicated for 30 min to fully drain the NA out of the LDH lattice. NA content was further quantified at 262 nm by a Jasco UV/Vis spectrometer (V-630, Jasco, Tokyo, Japan).

### 4.5. In Vitro Release Experiment

The NA release was completed using a paddle stirring procedure (DST-810 dissolution tester; Labfine, Seoul, Korea). We set the impeller speed at 50 rpm at 37 °C. Various samples that are in equivalence with 20 mg NA were mixed with 0.5 L release buffer. Samples were collected at pre-determined time points and were syringe-filtered using 450 nm PVDF filters (Thermo Scientific, Waltham, MA, USA). The NA was quantified as mentioned in Section 4.3. Each sample was measured in triplicate. Both gastric and intestinal buffers were used to simulate the fate of NA upon oral administration. The pH conditions of the release media were maintained at pH 1.2 and 6.8 to simulate the gastro-intestinal conditions, and experiments were conducted for 2 and 12 h, respectively.

### 4.6. MTT Analysis

MTT analysis was completed according to the previous protocol [87]. MTT is based on the reduction of the yellow tetrazolium salt MTT to a purple MTT formazan if the cells are viable with no toxicity. To demonstrate the cell viability of intact NA, pristine LDH and NA-LDH, MTT assay was performed in L929 cells under various concentrations (2.5 to 100 µg/mL) of particle after incubating for 24 h of treatment. For this, the normal fibroblast cell line (L929) was purchased from Korean Cell Line Bank and the cells were cultured in RPMI (Roswell Park Memorial Institute) 1640 medium (gibco. Ltd., New York, NY, USA) and maintained as reported before [87]. Various samples, i.e., NA, pristine LDH and NA-LDH in the concentration range of 2.5–100 μg/mL, and treated cells were kept in the CO_2_ incubator for 24 h. The cytocompatibility was detected by measuring the MTT formazan crystals by measuring absorbance at 540 nm using UV/Visible spectrophotometer (Multiskan FC, Thermo Scientific, Waltham, MA, USA).

## 5. Conclusions

In summary, we successfully intercalated NA molecules into a 2D lattice of LDH via the coprecipitation method in order to protect the NA very efficiently. After formulating the NA-LDH hybrid with Eudragit^®^ S100, which would be soluble in intestinal conditions, it would thereby be expected to have sustained NA release under gastrointestinal conditions. Additionally, the MTT assay revealed good biocompatibility of the hybrid drug, suggesting that it can be a potential drug delivery system without undesirable side effects, such as erythema and flushing, and requires administration of multiple doses and enhancing the bioavailability of NA.

## Data Availability

All the data mentioned can be found in the manuscript itself, and, if there is any additional requirement, please contact the corresponding author.

## Supplementary Materials

The following supporting information can be downloaded at: https://www.mdpi.com/article/10.3390/molecules27196439/s1, Figure S1. Scanning electron microscopy (SEM) image of Eudragit^®^ S100-coated NA-LDH; Figure S2. The particle size distribution by DLS: (a) pristine LDH, (b) NA-LDH and (c) Eudragit^®^ S100-coated NA-LDH; Figure S3. Plots of kinetic equation of (a) first-order kinetic model, (b) parabolic diffusion model, (c) modified Freundlich model and (d) Elovich model for the release of NA from NA-LDH, Eudragit^®^ S100-coated NA-LDH; Figure S4. TGA curves of (a) pristine LDH, (b) NA-LDH, (c) Eudragit^®^ S100-coated NA-LDH and (d) DTA curves of pristine LDH, NA-LDH and Eudragit^®^ S100-coated NA-LDH; Table S1. ICP analysis of pristine LDH and NA-LDH; Table S2. Characteristic bands in FT-IR spectra of intact NA, pristine LDH and NA-LDH. Table S3. Zeta potential analysis of pristine LDH, NA-LDH and Eudragit^®^ S100-coated NA-LDH; Table S4. Rate constants and r^2^ coefficients obtained from fitting analyses based on several kinetic equations.
