# Supplementary material for: pH-Responsive Inorganic/Organic Nanohybrids System for Controlled Nicotinic Acid Drug Release
Source: Molecules. 2022 Sep 29;27(19):6439. doi: 10.3390/molecules27196439 (PMC9571272; doi:10.3390/molecules27196439)
Supplement: Supplementary file 1 [file molecules-27-06439-s001.zip › molecules-1931130-supplementary.pdf]

molecules

Supplementary file

pH-Responsive Inorganic/Organic Nanohybrids System for Controlled Nicotinic Acid Drug Release

Seungjin Yu [1,2], Huiyan Piao [2], N. Sanoj Rejinold [2], Hanna Lee [2], Goeun Choi [1,2,3,*] and Jin-Ho Choy [2,4,5,6*]

[1] Department of Nanobiomedical Science and BK21 PLUS NBM Global Research Center for Regenerative Medicine, Dankook University, Cheonan 31116, Korea
[2] Intelligent Nanohybrid Materials Laboratory (INML), Institute of Tissue Regeneration Engineering (ITREN), Dankook University, Cheonan 31116, Korea
[3] College of Science and Technology, Dankook University, Cheonan 31116, Korea
[4] Division of Natural Sciences, the National Academy of Sciences, Seoul 06579, Korea.
[5] Department of Pre Medical Course, College of Medicine, Dankook University, Cheonan 31116, Korea
[6] International Research Frontier Initiative (IRFI), Institute of Innovative Research, Tokyo Institute of Technology, Yokohama 226-8503, Japan

* Correspondence: goeun.choi@dankook.ac.kr (G.C); jhchoy@dankook.ac.kr (J.-H.C.)

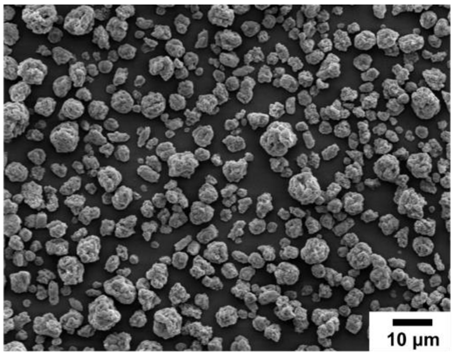

Figure S1. Scanning electron microscopy (SEM) image of Eudragit® S100-coated NA-LDH.

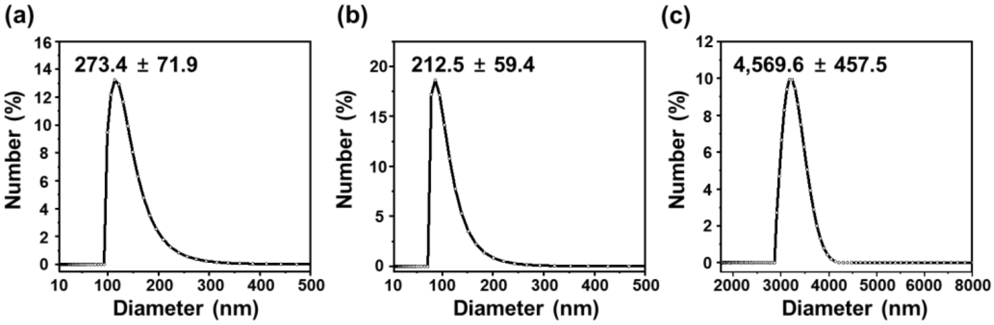

Figure S2. The particle size distribution by DLS: (a) pristine LDH, (b) NA-LDH and (c) Eudragit® S100-coated NA-LDH.

Table S1. ICP analysis of pristine LDH and NA-LDH.

	Mole of Zn	Mole of Al	Zn/Al molar ratio
pristine LDH	0.675	0.325	2.08
NA-LDH	0.668	0.332	2.01

Table S2. Characteristic bands in FT-IR spectra of intact NA, pristine LDH and NA-LDH.

Frequency assignment	Wavenumber (cm^{-1})		
	intact NA	pristine LDH	NA-LDH
O-H	3450	3400	3500-2500
C-H	3073, 2830	-	-
-COOH (Symmetric)	1320	-	-
$-COO^-$ (Symmetric)	-	-	1400
-COOH (Asymmetric)	1710	-	-
$-COO^-$ (Asymmetric)	-	-	1608
M-O, M-O-M	-	830-428	830-428
NO_3^-	-	1384	1384
-OH	-	1626	1693-1576

Table S3. Zeta potential analysis of pristine LDH, NA-LDH and Eudragit® S100-coated NA-LDH.

	Zeta potential (mV)
pristine LDH	40.9 ± 2.0
NA-LDH	-0.6 ± 1.0
Eudragit® S100-coated NA-LDH	-21.5 ± 0.6

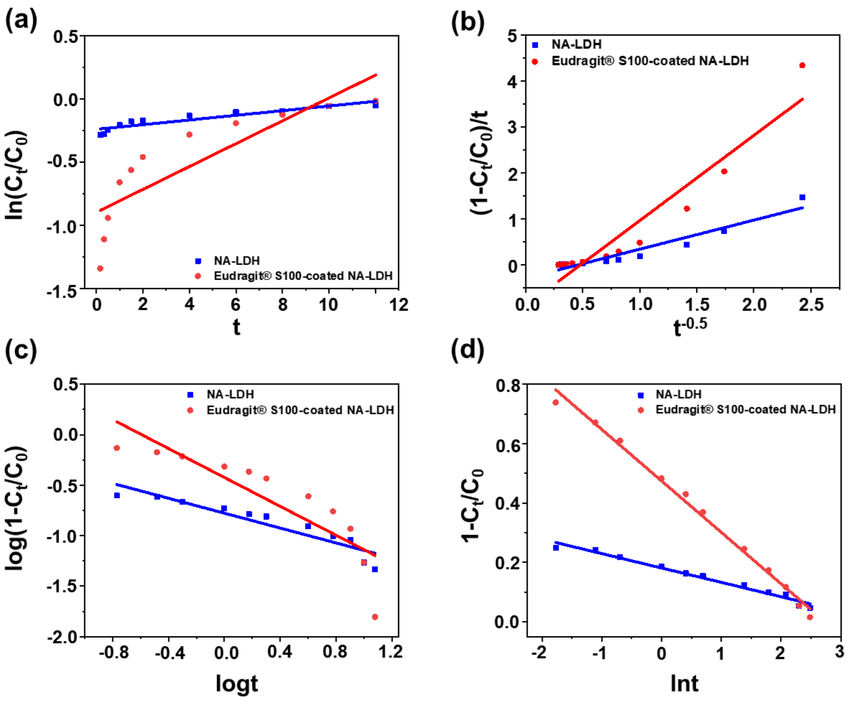

Figure S3. Plots of kinetic equation of (a) First-order kinetic model, (b) Parabolic diffusion model, (c) Modified Freundlich model, and (d) Elovich model for the release of NA from NA-LDH, Eudragit® S100-coated NA-LDH.

Table S4. Rate constants and r^2 coefficients obtained from fitting analyses based on several kinetic equations.

Kinetic model		NA-LDH	Eudragit® S100-coated NA-LDH
First order	kd	0.0185	0.0906
: $\ln(C_t/C_0) = -kdt$	r^2	0.8623	0.7298
Parabolic diffusion	kd	0.6329	1.8559
: $(1-C_t/C_0)/t = -kdt^{-0.5}+a$	a	-0.2942	-0.8946
	r^2	0.9238	0.9106
Freundlich	kd	-0.3684	-0.7153
: $\log(1-C_t/C_0) = \log(kd)+a\log t$	a	-0.7767	-0.4230
	r^2	0.8717	0.7515
Elovich	kd	-0.0484	-0.1732
: $1- C_t/C_0 = a\ln t + b$	a	0.1814	0.4744
	r^2	0.9774	0.9930

C_t = the amount of guest in the NA at t min;

C_0 = the amount of guest in the NA at 0 min;

kd = the rate constant of release, a, b, n = constant

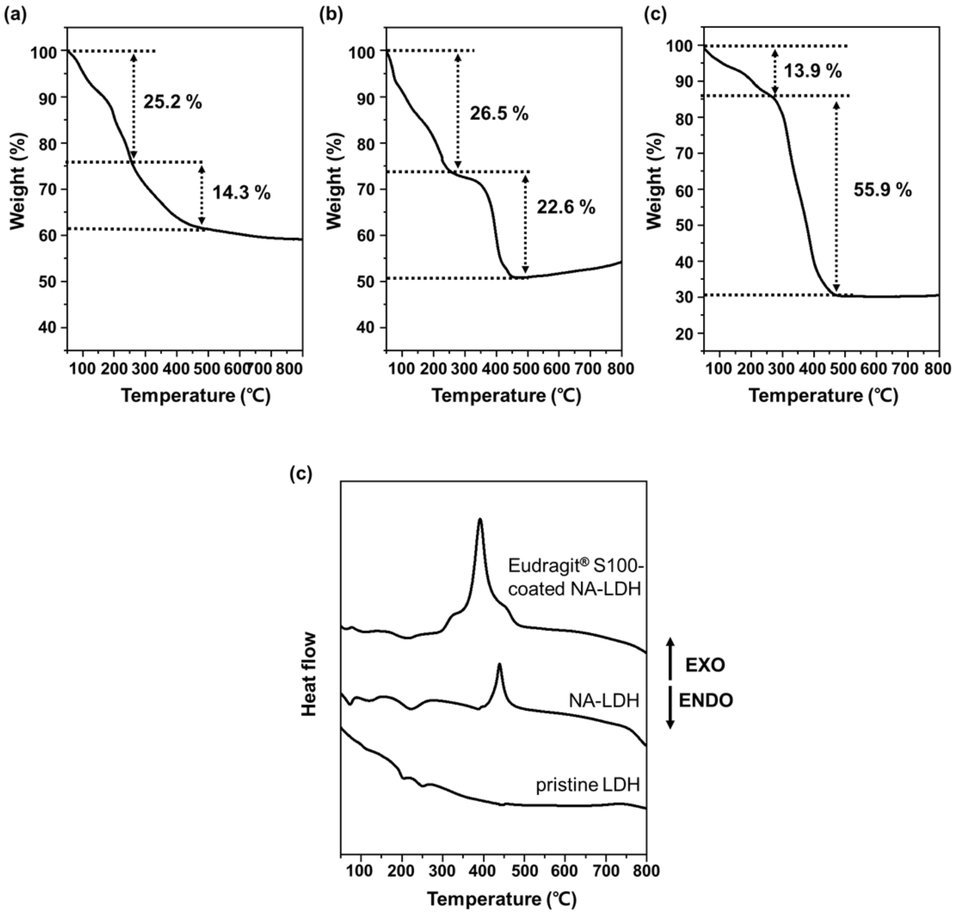

Figure S4. TGA curves of (a) pristine LDH, (b) NA-LDH, (c) Eudragit® S100-coated NA-LDH and (d) DTA curves of pristine LDH, NA-LDH and Eudragit® S100-coated NA-LDH.
